# Origin of Threshold Voltage Instabilities in Indium Oxide Transistors

**DOI:** 10.1021/acsami.5c20018

**Published:** 2026-02-23

**Authors:** Tzu-Jie Lin, Sheng-Chung Chen, Yung-Ting Lee, Sheng-Lun Cheng, Robert Tseng, Sung-Tsun Wang, Yu-Cheng Chang, Yi-Yu Pan, Chan-Yuen Chang, Tsung-Te Chou, Chia-Hsien Lin, Ching-Shun Ku, Chun-Liang Lin, Po-Tsun Liu, Hyungjin Kim, Der-Hsien Lien

**Affiliations:** † Institute of Electronics, 34914 National Yang Ming Chiao Tung University, Hsinchu 30010, Taiwan; ‡ College of Electrical and Computer Engineering, National Yang Ming Chiao Tung University, Hsinchu 30010, Taiwan; § Department of Applied Science, National Taitung University, Taitung 950309, Taiwan; ∥ Taiwan Instrument Research Institute, 87786 National Applied Research Laboratories, Hsinchu 300092, Taiwan; ⊥ Scientific Gear Service Co., Hsinchu 308003, Taiwan; # Department of Electrophysics, National Yang Ming Chiao Tung University, Hsinchu 30010, Taiwan; ∇ Department of Photonics, College of Electrical and Computer Engineering, National Yang Ming Chiao Tung University, Hsinchu 30010, Taiwan; ○ Department of Materials Science and Engineering, 26721 Yonsei University, Seoul 03722, Republic of Korea

**Keywords:** oxide semiconductors, In_2_O_3_, threshold voltage instability, charge exchange kinetics, surface oxygen, ultrathin films

## Abstract

Oxide semiconductors have gained substantial interest for their low-temperature processability, allowing for their integration as functional add-on device layers for advanced monolithic 3D integrated circuits (ICs). However, reliability issues, particularly under thermal, environmental, and electrical stresses, remain critical issues and require immediate solutions. This study investigates the instability of ultrathin In_2_O_3_ transistors, revealing that threshold voltage (*V*_T_) drifts arise from interactions between surface-adsorbed oxygen and the In_2_O_3_ channels. We show that the oxygen in the ambient atmosphere attached to the In_2_O_3_ surface plays a crucial role in modulating In_2_O_3_ conductivity, thereby governing *V*_T_. External perturbations such as ultraviolet (UV)/X-ray illumination, thermal annealing, and bias stress could alter this interaction of surface oxygen with ultrathin In_2_O_3_, leading to a *V*_T_ drift. Importantly, we propose a unified kinetic model that provides a generic physical description of *V*_T_ instabilities induced by these commonly observed factors. By characterizing time-dependent *V*_T_ instability, the model demonstrates that recovery dynamics exhibit identical behavior across all tested perturbations, indicating that the recovery process is independent of the initial stimulus. This study uncovers the surface oxygen as a critical factor affecting In_2_O_3_ transistor reliability, offering insights for designing oxide-based devices for advanced electronic and optoelectronic devices.

## Graphical abstract

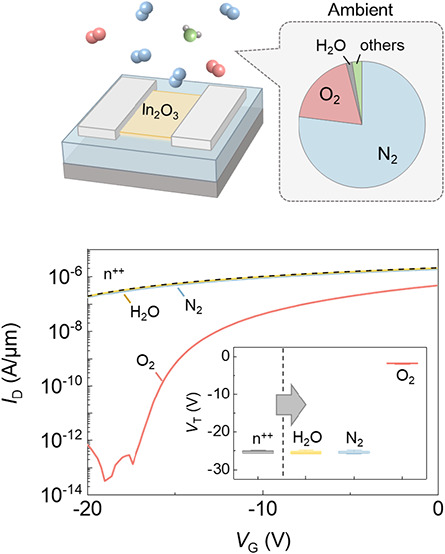

## Introduction

Atomically thin In_2_O_3_ has emerged as a promising material for advanced electronics due to its high mobility, ease of synthesis, and compatibility with monolithic 3D integration, making it well-suited for next-generation semiconductor technologies.^1–3^ Its tolerance to lattice disorder arises from the isotropic transport of s-orbital electrons, which maintain overlap even without high crystallinity.^4,5^ This unique feature enables In_2_O_3_ to achieve high mobility up to 100 cm^2^ V^–1^ s^–1^ in the amorphous phase,^6,7^ thereby circumventing crystallinity constraints and allowing for low-temperature synthesis. This intrinsic advantage meets the stringent thermal budget requirements of back-end semiconductor processes, making In_2_O_3_ highly compatible with heterogeneous integration into CMOS technologies.^8–10^

Ultrathin In_2_O_3_ transistors also exhibit notable tunability in their electronic properties, with the threshold voltage (*V*_T_) largely tunable by environmental and processing conditions. For example, recent studies have shown that the electronic characteristics of 2-nm-thick In_2_O_3_ can be substantially modulated through ultraviolet (UV) exposure, enabling a wide range of carrier concentrations that directly modulate *V*_T_.^11,12^ Similarly, annealing in nitrogen or oxygen environments can effectively shift the carrier density and electronic behavior of ultrathin films.^13,14^ However, this sensitivity also raises critical reliability concerns, leading to undesired *V*_T_ during the fabrication process;^15–17^ for example, pronounced *V*_T_ shifts are observed when dielectric layers are deposited on top of the transistors.^18,19^ It is also reflected in considerable reliability issues under electrical stress; for example, significant *V*_T_ drifts are observed under positive and negative bias stresses (PBS and NBS).^20–23^ Those instabilities are often attributed to oxygen-related trap states in the amorphous structure,^24–26^ while others suggest surface-adsorbed molecules are responsible for the conductivity changes,^27,28^ leaving the underlying mechanisms unresolved.

In this work, we investigate the mechanism of *V*_T_ instability in ultrathin In_2_O_3_ transistors. By excluding the effects of other air molecules, our results identify surface oxygen as the origin of the *V*_T_ drift, acting as an effective electron trap that modulates the conductivity of n-type In_2_O_3_. We show that external perturbations, such as high-energy illumination (UV and X-ray), thermal annealing, and gate bias stresses, induce *V*_T_ shifts by modulating the equilibrium and interfacial interactions between surface oxygen and the In_2_O_3_ channel. A kinetic model is proposed to quantify the *V*_T_ shifts and recovery dynamics. This study elucidates the surface phenomena in In_2_O_3_ transistors for further improving the device stability in next-generation electronic and optoelectronic applications.

## Results and Discussion

We first examine the *V*_T_ shifts of ultrathin In_2_O_3_ transistors under various external perturbations, including UV and X-ray exposure, high-temperature annealing, and electrical bias stress, followed by analysis of the subsequent *V*_T_ drift after these stimuli are removed. 2 nm In_2_O_3_ films were deposited on the Si/SiO_2_ substrate using atomic layer deposition (ALD), with the height confirmed by high-resolution transmission electron microscopy (HRTEM) and atomic force microscopy (AFM), as shown in Figure 1a. The optical microscope images of as-deposited In_2_O_3_ are also shown in Figure 1a. The *I*_D_–*V*_G_ transfer characteristics of In_2_O_3_ transistors exhibits on/off ratio of over 8 orders with *V*_D_ = 1 V, as illustrated in Figure 1b. Enhancement-mode operation is achieved in In_2_O_3_ transistors with 2 nm thickness, highlighting a key advantage of ultrathin films, whose conduction can be effectively modulated by the gate. The as-made device is operated in enhancement mode and could vary between the enhancement and depletion modes depending on the perturbation conditions. The *I*_D_–*V*_D_ output curves in Figure 1c exhibit clear saturation, with a maximum drain current of 125 μA/μm at *V*_D_ = 15 V.

**Figure 1:**
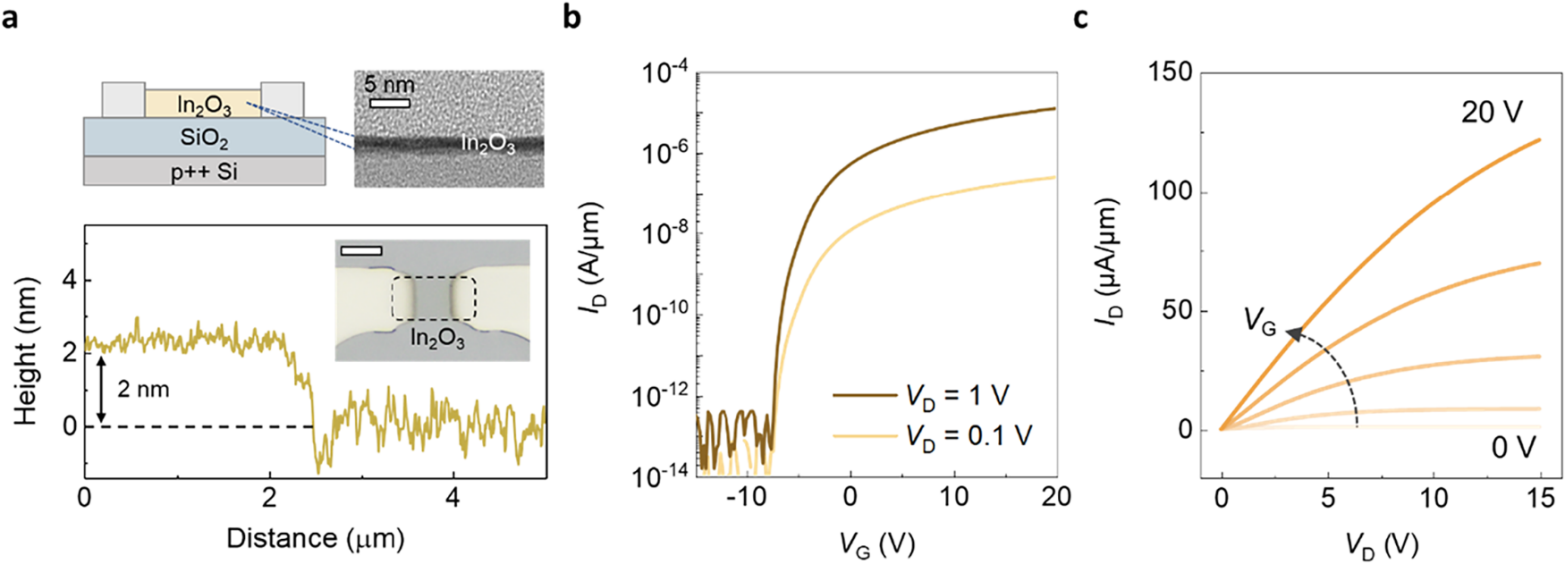
Material analysis and electrical characteristics of In_2_O_3_ transistors. (a) Schematic diagram of an In_2_O_3_ back-gate transistor and HRTEM image and AFM height profile of 2 nm In_2_O_3_. The inset shows an optical image of the ALD-deposited ultrathin In_2_O_3_ films. The scale bar is 5 μm. (b) *I*_D_–*V*_G_ characteristics of In_2_O_3_ back-gate transistors with different drain voltages. (c) *I*_D_–*V*_D_ characteristics of In_2_O_3_ back-gate transistors with different gate voltages.

UV exposure can induce conductivity changes in oxide semiconductors and has previously been shown to cause *V*_T_ shifts in In_2_O_3_ transistors.^29–32^ Figure 2a shows the transfer characteristics (*I*_D_–*V*_G_) of 2 nm In_2_O_3_ transistors exposed to UV irradiation (365 nm) from 1 to 600 s. The *V*_T_ of the transistors is extracted from the *I*_D_–*V*_G_, as shown in Figure 2b (extraction of *V*_T_ is described in Text S1). The In_2_O_3_ transistor shows a negative *V*_T_ shift upon UV illumination. As the UV exposure time extends, *V*_T_ shifts eventually reach a maximum Δ*V*_T_ of −10 V (*V*_T_ shift as a function of UV power density and exposure time in Figure S1). After the UV is turned off, the *V*_T_ gradually returns to its original value after days. Similar behavior has been observed in ZnO and SnO_2_, where UV exposure increases conductivity as photogenerated holes neutralize physiosorbed oxygen on the oxide surface,^33,34^ leading to its desorption and restoring free electrons to the channel. Upon cutting off the source, surface oxygen withdraws electrons from the n-type channel, reducing conductivity.^35^

**Figure 2:**
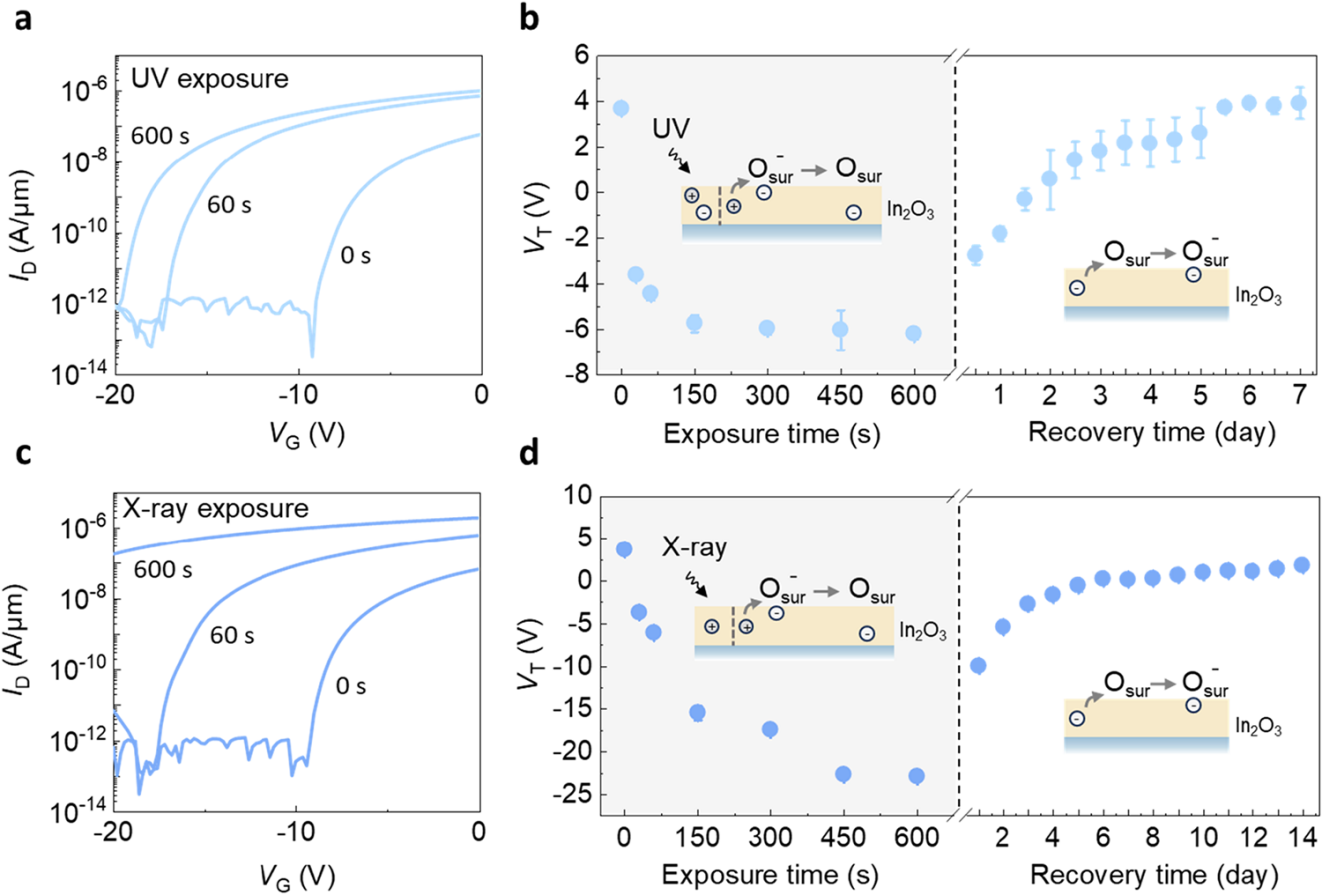
*V*_T_ shifts caused by UV and X-ray illumination and subsequent recovery in air. (a) The transfer characteristics *I*_D_–*V*_G_ plot of In_2_O_3_ transistor after different UV exposure time with *V*_D_ = 0.1 V. (b) *V*_T_ of a 2 nm In_2_O_3_ transistor with a channel width/length of 10/5 μm during UV exposure and recovery. *V*_T_ shifts more negatively with exposure time. *V*_T_ reverts to the initial value over time upon cutting off the UV source. (c) The transfer characteristics *I*_D_–*V*_G_ plot of In_2_O_3_ transistor after different X-ray exposure time with *V*_D_ = 0.1 V. (d) *V*_T_ shifts more negatively with exposure time. *V*_T_ reverts to its initial value over time while turning off the X-ray source.

To further verify the light-matter interaction mechanism, In_2_O_3_ was illuminated by an X-ray source (Mg K_α_; 1253.6 eV). With an increasing X-ray exposure time, 2 nm In_2_O_3_ transistors exhibit a negative *V*_T_ shift, as shown in Figure 2c,d. Resembling the trend observed under UV exposure, a maximum negative *V*_T_ shift (Δ*V*_T_ = −29 V) was observed for X-ray irradiation after an exposure time of over 600 s (*V*_T_ shift as a function of X-ray energy in Figure S2 and thickness dependence of In_2_O_3_ absorption spectra in Figure S3). Similar to the UV illumination, turning off the X-ray sources leads to a gradual recovery of *V*_T_ to its original value over several days (Figure 2b,d). Notably, *V*_T_ recovery occurs only when the devices are stored under ambient conditions, which will be discussed in detail later.

Under UV or X-ray exposure, the recovery of conductivity over several days is significantly longer than the minority carrier lifetime, suggesting that the effect is not governed by conventional recombination dynamics. Similar long-term light-induced conductivity changes have been previously observed in various metal-oxide semiconductors, including ZnO,^36^ TiO_2_,^37^ IGZO,^38,39^ and SnO_2_ ^40^ and are attributed to surface interactions involving the adsorption and desorption of ambient air molecules, particularly oxygen. These surface-related effects are further amplified in reduced-dimensional structures,^41^ such as nanowires,^42–46^ and nanorods,^47^ owing to their large surface-to-volume ratios.^36,48,49^ Among nanostructures, ultrathin films, especially at the quasi-2D limit, are most sensitive to surface effects due to their high surface-to-volume ratios. Despite this, surface effects in ultrathin In_2_O_3_ channels have been insufficiently explored.

To further examine the role of gas molecules in governing the transport characteristics of In_2_O_3_ transistors, annealing was conducted in both oxygen-rich and oxygen-scarce environments to regulate the total amount of oxygen on the In_2_O_3_ surface, as shown in Figure 3. To create oxygen-scarce environments, In_2_O_3_ transistors were annealed at 150 °C in nitrogen. The *I*_D_–*V*_G_ in Figure 3a shows that the curves shift negatively with increasing annealing time in a nitrogen environment, with a maximum negative Δ*V*_T_ of −22 V. The *V*_T_ recovers to the original value when the device is cooled to room temperature in normal ambient, as illustrated in Figure 3b. The result indicates that annealing in a N_2_ environment reduces surface oxygen concentration, suppressing electron trapping effects and consequently increasing conductivity, leading to a negative *V*_T_ shift. In contrast, oxygen-rich annealing produces opposite results. When annealed in oxygen at 150 °C, In_2_O_3_ transistors exhibit a positive *V*_T_ shift with an increasing annealing time, as shown in Figure 3c. The *I*_D_–*V*_G_ shows a maximum positive Δ*V*_T_ of 1 V, along with the subsequent recovery of *V*_T_ after cooled down in the ambient, as illustrated in Figure 3d. In this case, overall surface oxygen concentration is increased, leading to a higher charged surface oxygen concentration and a positive *V*_T_ shift.

**Figure 3:**
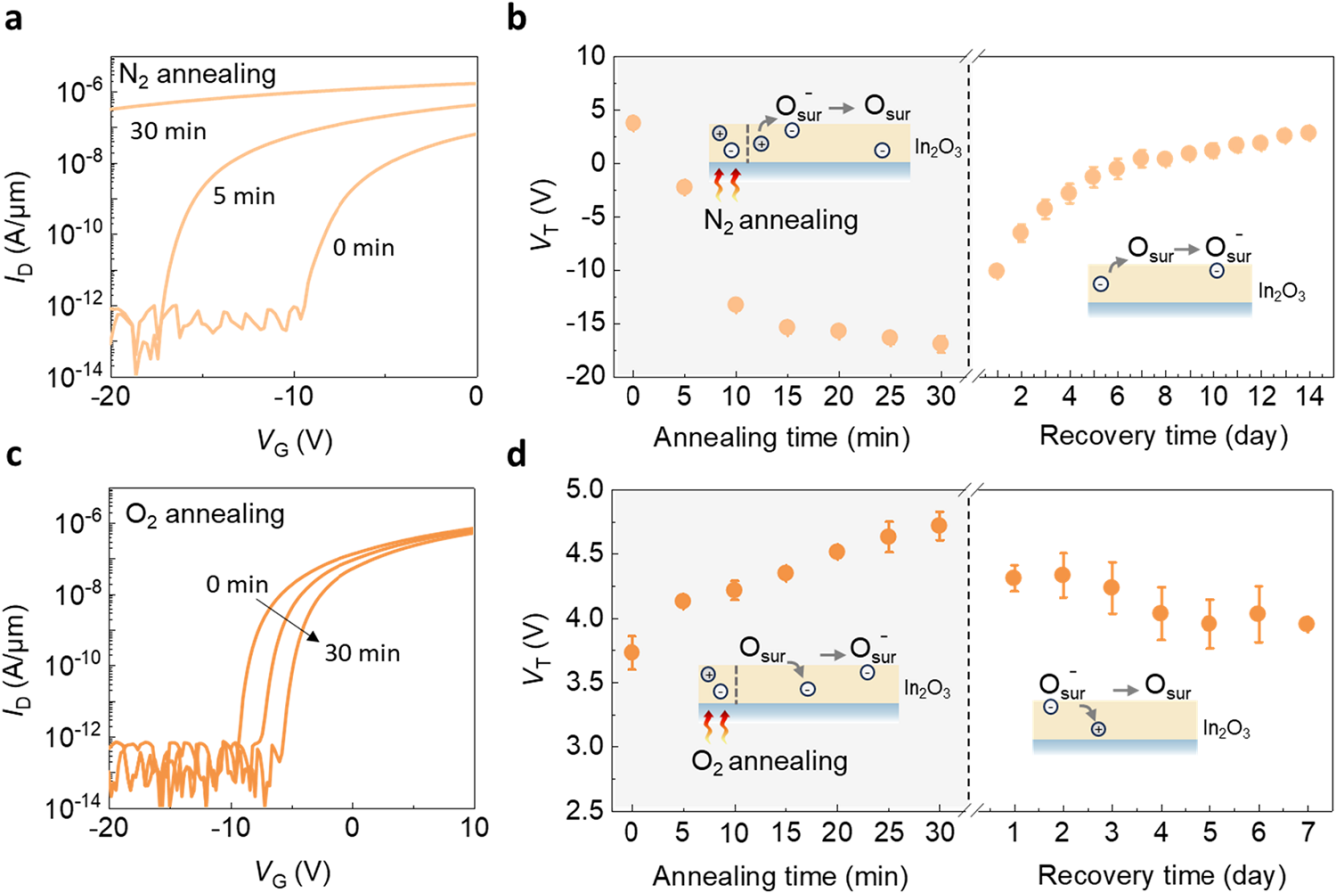
*V*_T_ shifts caused by N_2_ and O_2_ annealing and subsequent recovery in air. (a) The transfer characteristics *I*_D_–*V*_G_ plot of In_2_O_3_ transistor after different N_2_ annealing time with *V*_D_ = 0.1 V. (b) *V*_T_ shifts more negatively with the annealing time. *V*_T_ reverts to the initial value over time when the device is placed in the air. (c) The transfer characteristics *I*_D_–*V*_G_ plot of In_2_O_3_ transistor after different O_2_ annealing time with *V*_D_ = 0.1 V. (d) *V*_T_ shifts more positively with annealing time. *V*_T_ reverts to the initial value over time when the device is placed in the air.

To clarify the role of electrons and holes in In_2_O_3_, gate bias was applied to modulate the carrier density in In_2_O_3_.^19,50^ When a negative gate bias (*V*_G_ = −15 V) was applied, *V*_T_ shows a negative shift with a maximum negative Δ*V*_T_ of −4.8 V, as shown in Figure 4a,b. Since In_2_O_3_ is an n-type semiconductor, a negative gate bias increases the minority carrier density (holes). Although hole-branch current is not observed, likely due to low mobility or high contact resistance for holes, the increased hole density under negative gate bias facilitates charged surface oxygen neutralization. This process leads to a negative *V*_T_ shift, similar to the effect of UV/X-ray exposure and oxygen-scarce annealing. Conversely, applying a positive gate bias of *V*_G_ = 15 V resulted in a positive *V*_T_ shift, as shown in Figure 4c,d. This positive *V*_T_ shift is due to a reduction in hole density caused by the gate bias, which promotes electron transfer from In_2_O_3_ to surface oxygen and lowers electrical conductivity. Since holes are minority carriers in n-type In_2_O_3_, this reduction has a limited effect on the balance of the surface oxygen, resulting in a slight positive *V*_T_ shift.

**Figure 4:**
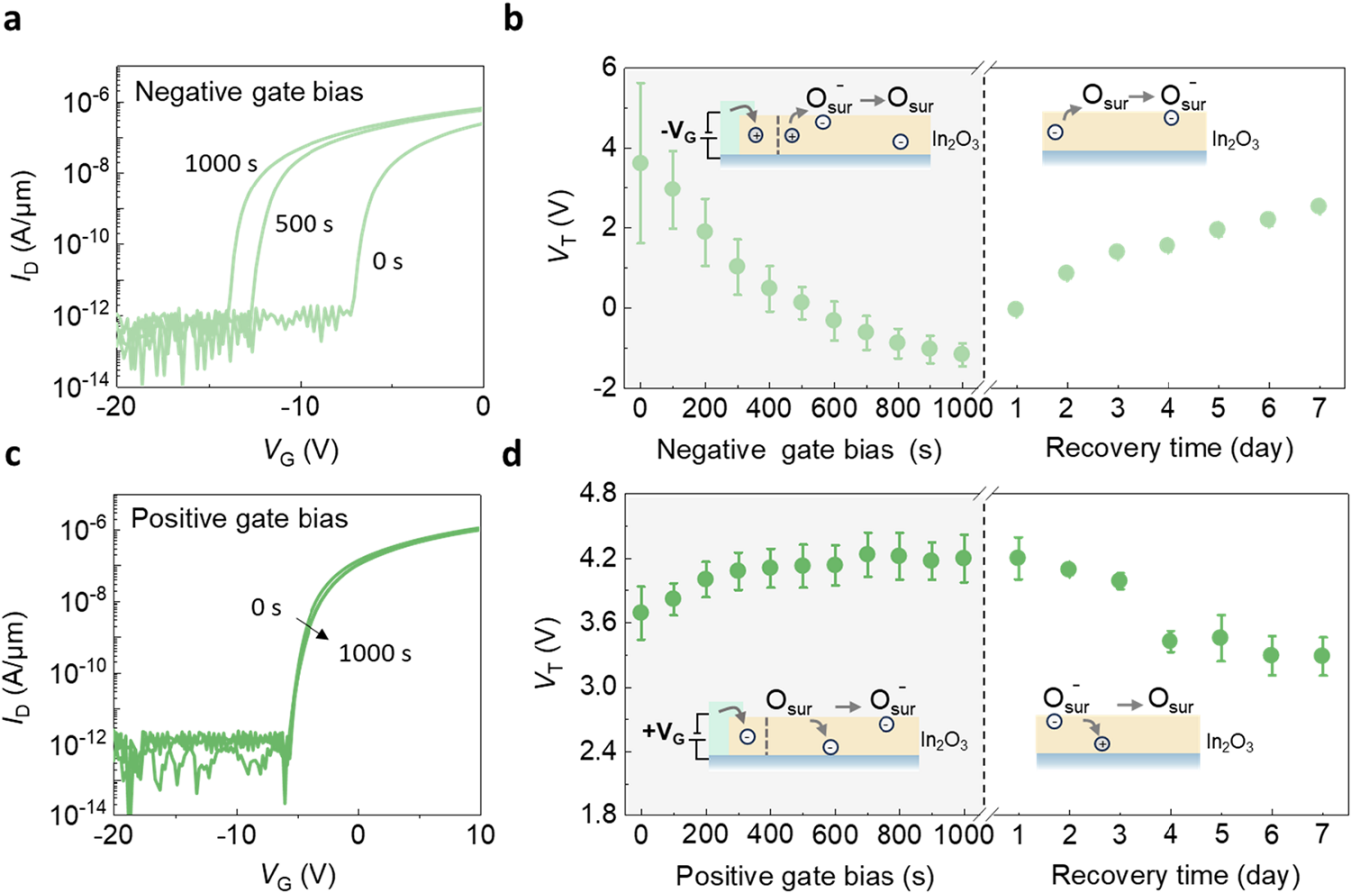
*V*_T_ shifts caused by negative and positive gate bias and subsequent recovery in air. (a) The transfer characteristics *I*_D_–*V*_G_ plot of In_2_O_3_ transistor after different negative gate bias time with *V*_G_ = −15 V. (b) *V*_T_ shifts more negatively with a negative gate bias time. *V*_T_ reverts to its initial value over time when cutting off the bias source. (c) The transfer characteristics *I*_D_–*V*_G_ plot of In_2_O_3_ transistor after different positive gate bias time with *V*_G_ = 15 V. (d) *V*_T_ shifts more positively with positive gate bias time. *V*_T_ reverts to its initial value over time when cutting off the bias source.

While oxygen is widely recognized as the primary ambient species affecting the conductivity of oxide materials, the effects of other air molecules remain unclear. To investigate their impact on ultrathin In_2_O_3_, we examine the *V*_T_ drift by exposing a degenerated In_2_O_3_ channel to N_2_, O_2_, H_2_O, three major constituents of ambient air. Among these, only oxygen exposure leads to *V*_T_ recovery, while no significant change is observed under nitrogen or water vapor, as shown in Figure 5a,b, confirming the dominant role of oxygen. Density functional theory (DFT) simulations also support the strong charge exchange between surface oxygen and In_2_O_3_ (Figures S4–S6). The results show that with the adsorption of oxygen on the In_2_O_3_ surface (either in the form of mono oxygen or oxygen molecules), the Fermi level drops to the bottom of the bandgap, indicating a transfer of electrons from the In_2_O_3_ to oxygen.^51–54^

**Figure 5:**
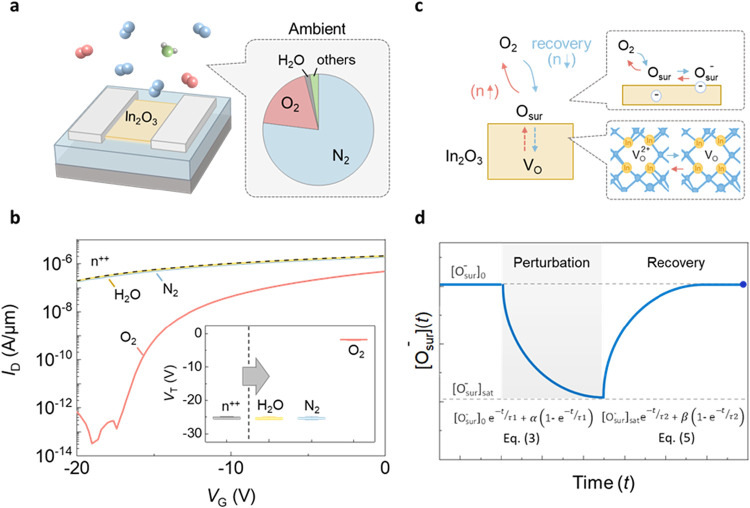
Charge exchange kinetics of oxygen in an ambient environment. (a) Schematic diagram of an In_2_O_3_ back-gate transistor exposed to air, consisting of 78% nitrogen, 21% oxygen, and water vapor. (b) Degenerated In_2_O_3_ channels were exposed to N_2_, O_2_, and H_2_O, revealing that only oxygen exposure induces *V*_T_ recovery, whereas no significant change is observed under nitrogen or water vapor. (c) Schematic illustration of charge exchange between In_2_O_3_ and oxygen species. Under external perturbations, surface-charged oxygen undergoes neutralization by holes, leading to a decrease in concentration until it reaches a steady state. Upon removal of the perturbations, oxygen recaptures electrons and diffuses into In_2_O_3_ to refill the oxygen vacancy, resulting in a gradual increase in its concentration. (d) The [O_sur_^–^]­(*t*) under external perturbations is described by eq 3, and the recovery dynamics is described by eq 5. Note that the equation parameters are lumped into coefficients α, β, τ_1_, and τ_2_ for simplicity.

Here, a kinetic model is developed, where the whole charge exchange kinetics associated with the surface oxygen is governed by three processes: (i) transport of oxygen molecules to the In_2_O_3_ surface, (ii) adsorption and desorption of oxygen at the In_2_O_3_ surface, and (iii) charge exchange between oxygen and In_2_O_3_. The diffusion rate of oxygen from the atmosphere to the surface of In_2_O_3_ is determined by Henry’s law *F* = *h*_g_(*C*_g_ – *C*_s_), where *h*_g_ is the gas phase mass-transfer coefficient, *C*_g_ is the oxygen concentration of air, and *C*_s_ is the oxygen concentration on the surface. In ambient conditions, transport of oxygen from the atmosphere to the In_2_O_3_ surface occurs within milliseconds (Text S2). While oxygen arrives at the surface, the physiosorbed rate is determined by *R* = *FS*, where *F* is the flux of oxygen and *S* is the sticking coefficient (fraction of gas molecules that adsorb upon hitting the surface). In ambient conditions, oxygen adsorption and desorption reach an equilibrium within seconds (Text S3). Once the adsorption and desorption dynamics of oxygen reach equilibrium, a stable charge exchange pathway is established between the oxygen species and the In_2_O_3_ channel. The total oxygen concentration is defined as [O_sur_^–^]_tot_ = [O_sur_] + [O_sur_^–^], where [O_sur_] represents the concentration of neutral oxygen species, [O_sur_^–^] refers to the concentration of oxygen that withdraws electrons from the In_2_O_3_.

As the observed *V*_T_ shift dynamics are much slower than the first two processes, the kinetics is predominant by the charge exchange that occurs between the oxygen and In_2_O_3_, which can be described by the following first-order reversible reactions

1 $$O_{\mathrm{sur}}^{-}+h^{+}\begin{array}{l} k_{1}\\ ⇌\\ k_{2} \end{array}O_{\mathrm{sur}}$$

2 $$O_{\mathrm{sur}}+e^{-}\begin{array}{l} k_{3}\\ ⇌\\ k_{4} \end{array}O_{\mathrm{sur}}^{-}$$

where the constant *k*_1_ and *k*_2_ are the forward and reverse rate constants of eq 1, respectively, while *k*_3_ and *k*_4_ are the forward and reverse rate constants of eq 2, respectively. For eq 1, electrons exchange from oxygen to In_2_O_3_, and the oxygen restores the neutral state. For eq 2, electrons transfer to the oxygen from the In_2_O_3_ to oxygen, so the oxygen is charged. According to the two equations, we obtain the general solution for [O_sur_^–^] as a function of time (details of derivation in Text S4)­

3 $$\left[O_{\mathrm{sur}}^{-}\right]\left(t\right)={\left[O_{\mathrm{sur}}^{-}\right]}_{0}e^{-\left(k_{1}\delta p+k_{2}+k_{3}\delta n+k_{4}\right)t}+\frac{\left(k_{2}+k_{3}\delta n\right){\left[O_{\mathrm{sur}}\right]}_{\mathrm{tot}}}{k_{1}\delta p+k_{2}+k_{3}\delta n+k_{4}}\left(1-e^{-\left(k_{1}\delta p+k_{2}+k_{3}\delta n+k_{4}\right)t}\right)$$

where δ*n* and δ*p* are excess electron and hole densities in the nonequilibrium state, which is determined by the external perturbations. Note that all perturbations operate under high-level injection conditions, where carrier density is governed by δ*n* and δ*p*, as they surpass the equilibrium electron (*n*_0_) and hole (*p*_0_) densities. [O_sur_^–^]_0_ is the initial surface-charged oxygen concentration under thermal equilibrium in the ambient environment. Equation 3 shows that [O_sur_^–^]­(*t*) decays exponentially over time when the first term dominates, as observed under UV illumination, X-ray illumination, N_2_ annealing, and negative bias stressing, as illustrated in Figure 5c,d (Fourier-transform infrared (FTIR) spectra of surface-adsorbed oxygen on In_2_O_3_ films in Figure S7). The equation shows that [O_sur_^–^] increases with an exponential saturation trend when the second term dominates, as seen in the cases of O_2_ annealing and positive bias stressing. We then use the kinetic model to examine the dynamics of the *V*_T_ shift under various perturbations shown in this study. Each stimulus drives δ*n* and δ*p* toward a new equilibrium through the respective mechanisms described above. The altered carrier densities in the nonequilibrium state shift the [O_sur_^–^] concentration toward a new equilibrium, with the rebalancing time dictated by both δ*n* and δ*p*, and the reaction rate constants. As oxygen extracts an electron from In_2_O_3_, it effectively acts as an acceptor dopant. Consequently, changes in [O_sur_^–^] modulate the carrier concentration of In_2_O_3_. According to the Drude model, variations in the carrier concentration induced by [O_sur_^–^] directly correspond to a linear shift in *V*_T_. By fitting the *V*_T_-time curves using eq 3, the time constants required to achieve the rebalanced [O_sur_^–^] under different stimuli can be obtained, as shown in Figure 6a–d (The parameters associated with each specific perturbation are summarized in Figure S8 and Table S1. Post-perturbation mobility change is also shown in Figure S9).^55,56^

**Figure 6:**
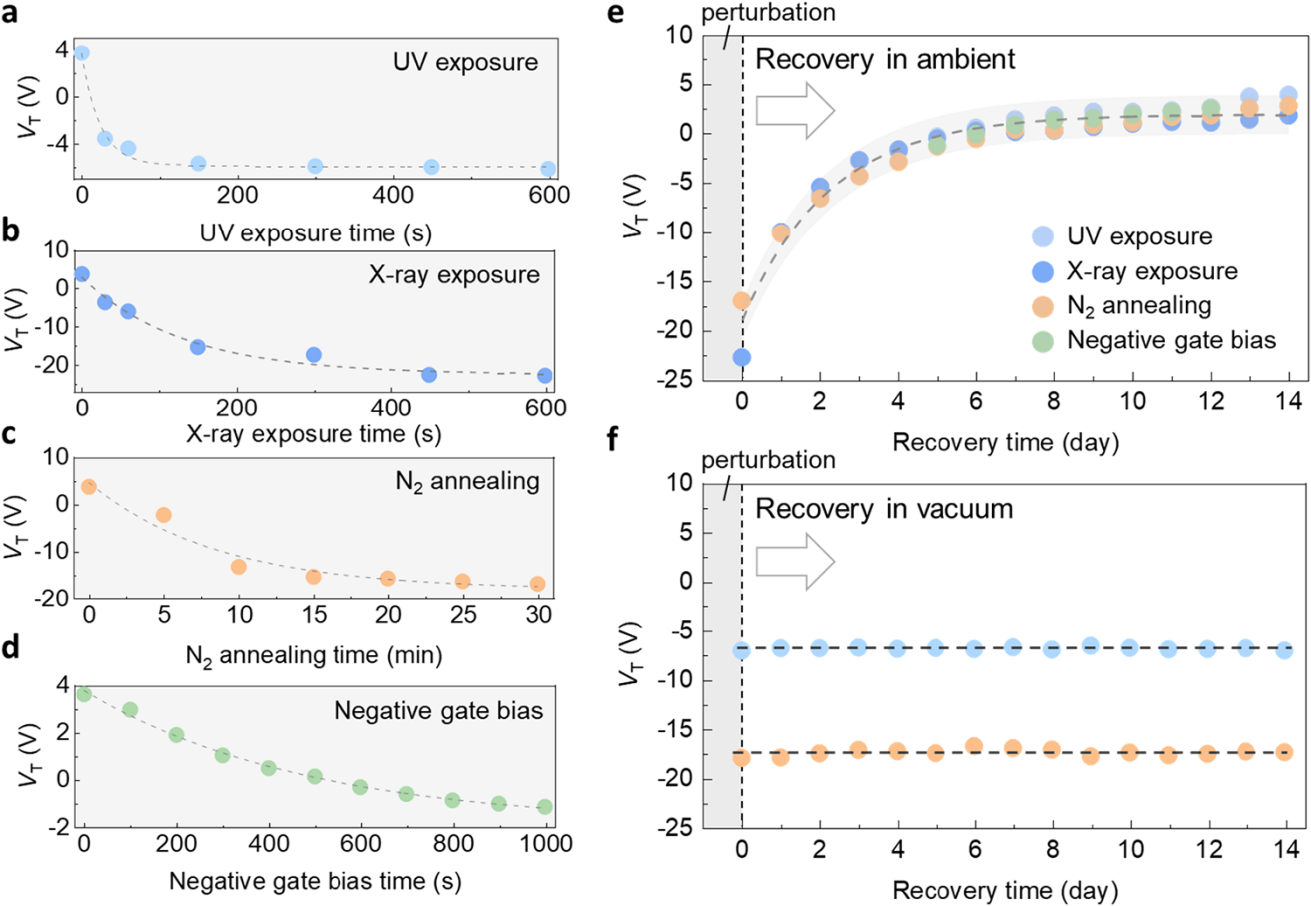
First-order reversible equation fitting of In_2_O_3_ oxygen charge exchange with different treatment (a) UV exposure, (b) X-ray exposure, (c) N_2_ annealing, and (d) Negative gate bias. The respective time constants are 23 s, 130 s, 8 min, and 480 s for the above perturbations, respectively. (e) A first-order reversible equation is employed to model the recovery mechanism by aligning the initial points of the various treatments. The time constant is about 2 days. (f) The *V*_T_ in vacuum exhibits no recovery due to the minimal availability of oxygen. The vacuum environment prevents oxygen desorbed from the surface from returning to the In_2_O_3_ surface for charge exchange, so *V*_T_ remains at its original value after UV exposure and N_2_ annealing.

The same kinetic model can also be applied to the recovery dynamics. The stimuli rebalance the [O_sur_^–^], which reaches the saturation value [O_sur_^–^]_sat_

4 $${\left[O_{\mathrm{sur}}^{-}\right]}_{\mathrm{sat}}=\frac{\left(k_{2}+k_{3}\delta n\right)}{k_{1}\delta p+k_{2}+k_{3}\delta n+k_{4}}{\left[O_{\mathrm{sur}}\right]}_{\mathrm{tot}}$$

Upon removal of the perturbations, [O_sur_^–^] begins to restore the initial value from [O_sur_^–^]_sat_. Meanwhile, with the removal of the perturbations, electron and hole densities return to their thermal equilibrium values *n*_0_ and *p*_0_, respectively. The variance of [O_sur_^–^] can be obtained from the same kinetic model

5 $$\left[O_{\mathrm{sur}}^{-}\right]\left(t\right)={\left[O_{\mathrm{sur}}^{-}\right]}_{\mathrm{sat}}e^{-\left(k_{1}p_{0}+k_{2}+k_{3}n_{0}+k_{4}\right)t}+\frac{\left(k_{2}+k_{3}n_{0}\right){\left[O_{\mathrm{sur}}\right]}_{\mathrm{tot}}}{k_{1}p_{0}+k_{2}+k_{3}n_{0}+k_{4}}\left(1-e^{-\left(k_{1}p_{0}+k_{2}+k_{3}n_{0}+k_{4}\right)t}\right)$$

The equation indicates that when the recovery time is sufficiently long, [O_sur_^–^]­(*t*) reverts to its initial value [O_sur_^–^]_0_, as all cases eventually restore their initial equilibrium under identical ambient conditions (Text S5). Therefore, when overlaying the recovery curves, they closely align, as shown in Figure 6e (The parameters associated with each recovery are summarized in Figure S8 and Table S1. For simplicity, the parameters are lumped into coefficients α, β, τ_1_, and τ_2_ in eqs 3 and 5.). Additionally, fitting these curves with eq 5 yields similar recovery time constants (∼2 days) across different cases, confirming a consistent recovery mechanism. Notably, the intrinsic minority carrier lifetime of In_2_O_3_ typically ranges from tens to hundreds of nanoseconds and is expected to be even shorter in amorphous films due to higher trap densities.^57–59^ In contrast, the observed conductivity recovery in this study occurs over days, far exceeding the minority carrier lifetime. This difference arises because the extended perturbation time provides a sufficient nonequilibrium window to activate surface oxygen adsorption/desorption kinetics. Furthermore, the long recovery time could arise from two possible pathways: charge exchange between physiosorbed oxygen and the In_2_O_3_ channel, or diffusion of surface oxygen into the bulk, accompanied by vacancy filling, as illustrated in Figure 5c (Thickness dependence of In_2_O_3_ devices postperturbation in Figures S10–S12). Both mechanisms could explain the observed recovery behavior; however, the exact mechanism of the underlying charge exchange process remains to be clarified.

To further examine the pressure effect, the transistors were placed in a vacuum environment following external perturbations, as shown in Figure 6f. Different from previous results in the ambient, the negative *V*_T_ shift caused by the UV exposure and N_2_ annealing treatment shows no recovery phenomenon when the device is stored in a vacuum. The *V*_T_ shift remained after 14 days. The results indicate that the neutralization of oxygen [O_sur_^–^ → O_sur_] could cause a desorption process of surface oxygen.^19,40,41,43,44,48^ When the In_2_O_3_ surface is exposed to a vacuum, in the absence of an additional oxygen supply, the charge exchange process described in eq 2 is inhibited. Consequently, this leads to a significant reduction in [O_sur_^–^]_tot_, weakening the contribution of the second term of the exponential in eq 5, which governs the recovery mechanism. This controllable recovery phenomenon offers a unique opportunity to emulate synaptic plasticity and multi-scale temporal information processing in neuromorphic systems, as explored in recent studies.^60,61^

## Conclusions

In summary, this research elucidates the underlying mechanism of electrical instabilities of ultrathin In_2_O_3_ devices caused by external perturbations. We show that the modulation of *V*_T_ is primarily attributed to the charge exchange between surface oxygen and In_2_O_3_. A key contribution of this study is the development of a unified kinetic model that captures the *V*_T_ recovery dynamics following illumination, annealing, and electrical perturbations within a generic model. The *V*_T_ recovery at room temperature indicates that the charge exchange of surface oxygen, along with the adsorption and desorption processes, is the primary reason influencing carrier concentration, rather than the formation of oxygen vacancies or defects. The results indicate that adding a caping layer on In_2_O_3_ could effectively prohibit the exchange of surface oxygen, thus improving the reliability, explaining the result reported previously.^18,19^ This work also supports that surface functionalization^6^ could potentially passivate the surface and suppress surface interaction, thereby improving device stability. This passivation mechanism is expected to be universally applicable across various oxide semiconductors, offering critical insights into developing advanced strategies to improve the reliability of oxide-based electronics and optoelectronics.

## Experimental Section

### In_2_O_3_ Growth and Transistor Fabrication

2 nm-thick In_2_O_3_ thin films were deposited on SiO_2_/p^+2^Si substrates by ALD, forming a basic metal–semiconductor-oxide (MOS) structure. The 30 nm-thick SiO_2_ layer serves as the dielectric, with heavily phosphorus-doped Si (p^+2^Si) as the back gate, and the ultrathin In_2_O_3_ layer exposed in air. The active area was then defined by photolithography, followed by etching of In_2_O_3_ using a diluted solution of HCl. 30 nm-thick nickel was deposited on In_2_O_3_ to form the source-drain metal electrodes. The thickness of the as-deposited In_2_O_3_ was measured using an ellipsometer, AFM, and HRTEM.

### Transistor Characterizations

The In_2_O_3_ devices were exposed to UV and X-ray illumination. The 365 nm UV light source is LED, and the X-ray light source is 1253.6 eV with Mg K_α_, and the other one is 20–80 keV with a Teresa nano-CT system (Excillum NanoTube N3). The devices were also exposed for 600 s. The In_2_O_3_ devices were placed in a customized chamber with two gas inlets. Then, the devices were annealed in O_2_ and N_2_ with a 1 L/min gas flow at 150 °C. The pressure was kept at about 1 atm. The negative and positive gate bias were generated by Keysight Agilent B2902B source at room temperature. The devices were stressed by ±15 V for 1000 s under atmospheric conditions. The electrical characteristics were measured using Keysight Agilent B2902B source at room temperature, in the absence of light, under atmospheric conditions.

### DFT Calculation

First-principles calculations of In_2_O_3_ materials were performed using OpenMX (v3.9.9) within the framework of density functional theory (DFT). The computations utilized the generalized gradient approximation (GGA), norm-conserving pseudopotentials, and optimized pseudoatomic basis functions.^62–66^ The exchange-correlation interactions were treated using the Perdew–Burke–Ernzerhof (PBE) functional within GGA.^67^ The optimized radial functions used were In-s3p2d2 for indium and O-s2p2d1 for oxygen, respectively. The criterion for force convergence was set to 1 × 10^–4^ hartree/bohr, and the electronic self-consistent field convergence criterion was set to 1 × 10^–9^ hartree. The energy cutoff was 400 Ry for all DFT calculations. The DFT model comprises a corundum-type In_2_O_3_ layer, representing an amorphous In_2_O_3_ layer. The optimized In_2_O_3_ film with an adsorbed oxygen atom [O_sur_] consisted of 31 atoms, including 12 indium and 19 oxygen atoms. The *k*-point grid was 6 × 6 × 1 for the In_2_O_3_ film.

### The In_2_O_3_ Annealing Environment

The oxygen-rich environment refers to pure ambient (99.999% purity) O_2_ at 1 atm. The oxygen-scarce environment refers to a high-purity N_2_ ambient (99.999% purity), where the oxygen partial pressure is minimized. Quantitatively, the oxygen level in the N_2_ environment is estimated to be less than 10 ppm, which is several orders of magnitude lower than that in the O_2_ environment.

## Supplementary Material



## Notes

Extraction of threshold voltage; a contour plot of *V*_T_ variation with UV power density and exposure time; a simulation of 2 nm In_2_O_3_ transmission rate; thickness dependence of In_2_O_3_ absorption spectra; a DFT simulation of intrinsic In_2_O_3_ surface and surface oxygen adsorbed on In_2_O_3_ surface; time for oxygen diffuse to In_2_O_3_ surface; time for oxygen adsorb on In_2_O_3_ surface; general solution of two first-order reversible reaction; FTIR transmittance spectrum shows the surface-adsorbed oxygen on In_2_O_3_ films; surface oxygen dynamics under four different perturbation conditions; comparison of parameters under different perturbation and their recovery; the field effect mobility of ultrathin In_2_O_3_ transistors as a function of *V*_T_ tuned by the proposed methods; demonstrating the charged surface oxygen concentration equivalence of prestimulus and postrecovery states; the transfer characteristics *I*_D_–*V*_G_ plot of 2 and 4 nm In_2_O_3_ transistor after different perturbation (PDF)
