# Supplementary material for: Origin of Threshold Voltage Instabilities in Indium Oxide Transistors
Source: ACS Appl Mater Interfaces. 2026 Feb 23;18(10):15676–85. doi: 10.1021/acsami.5c20018 (PMC13006957; doi:10.1021/acsami.5c20018)
Supplement: Supplementary file 1 [file am5c20018_si_001.pdf]

Supporting Information

Origin of Threshold Voltage Instabilities in Indium Oxide Transistors

*Tzu-Jie Lin[1,2], Sheng-Chung Chen[1], Yung-Ting Lee[3], Sheng-Lun Cheng[1], Robert Tseng[1,2], Sung-Tsun Wang[1], Yu-Cheng Chang[1], Yi-Yu Pan[1,2], Chan-Yuen Chang[4], Tsung-Te Chou[4], Chia-Hsien Lin[5], Ching-Shun Ku[5], Chun-Liang Lin[6], Po-Tsun Liu[7], Hyungjin Kim[8], Der-Hsien Lien[1,2]**

[1]Institute of Electronics, National Yang Ming Chiao Tung University, Hsinchu 30010, Taiwan

[2]College of Electrical and Computer Engineering, National Yang Ming Chiao Tung University, Hsinchu 30010, Taiwan

[3]Department of Applied Science, National Taitung University, Taitung 950309, Taiwan

[4]Taiwan Instrument Research Institute, National Applied Research Laboratories, Hsinchu 300092, Taiwan

[5]Scientific Gear Service Co., Hsinchu 308003, Taiwan

[6]Department of Electrophysics, National Yang Ming Chiao Tung University, Hsinchu 30010, Taiwan

[7]Department of Photonics, College of Electrical and Computer Engineering, National Yang Ming Chiao Tung University, Hsinchu 30010, Taiwan

[8]Department of Materials Science and Engineering, Yonsei University, Seoul 03722, Republic of Korea

[*] Address correspondence to dhlien@nycu.edu.tw

Text S1. Extraction of threshold voltage

Threshold voltage (V_T) is extracted under linear regime ($V_D \ll V_G$ - V_T) by $I_{DS} = \frac{W}{L}\mu_{FE}C_{OX}(V_G - V_T)V_{DS}$, where W is the channel width, L is the channel length, μ_{FE} is the electron mobility and C_{OX} is the capacitance of the gate oxide. The resulted V_T was determined by linear extrapolation, which was conducted by plotting I_D versus V_G, extrapolating from maximum transconductance (g_m) to $I_D = 0$ and adding $\frac{V_D}{2}$ to obtain the intercept at V_G axis.

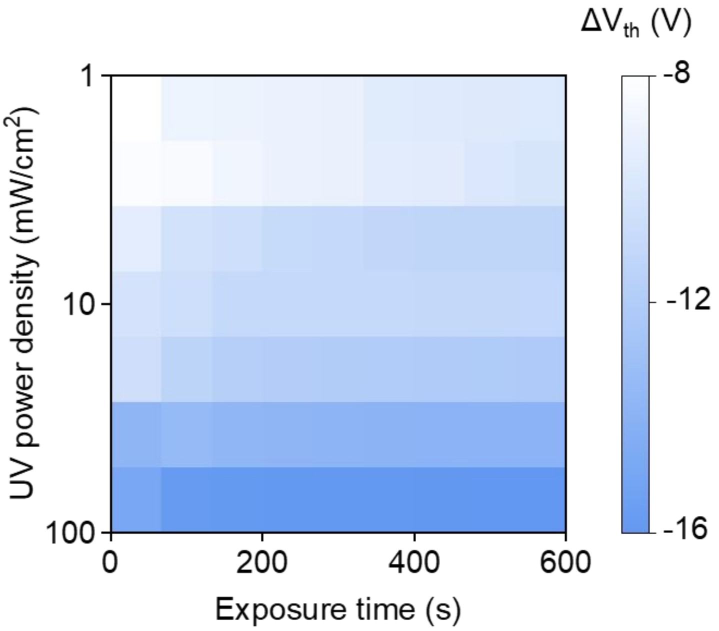

Figure S1. A contour plot of V_T variation with UV power density from 1 mW cm^{-2} to 100 mW cm^{-2} for exposure times from 0 s to 600 s.

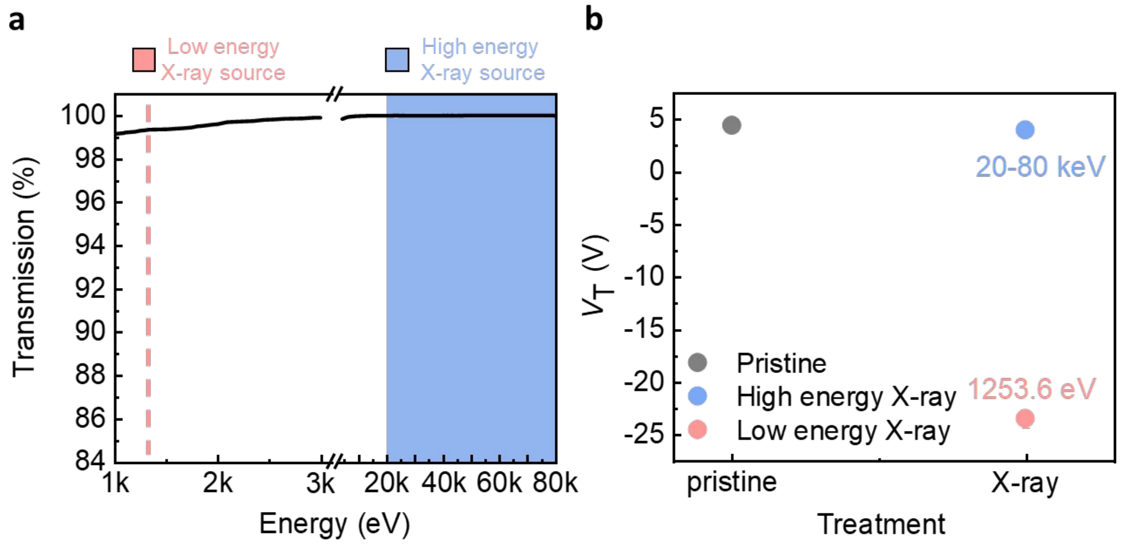

Figure S2. (a) A simulation of 2 nm In_2O_3 transmission rate from 1 to 80 keV. The black line represents the simulation results, while the red line corresponds to the low-energy X-ray (1253.6 eV) data from the experiment. The blue-shaded region (20 - 80 keV) indicates the high-energy X-ray range used in the experiment. (b) The behavior of V_T varies across different experimental conditions. Under low-energy X-ray exposure, V_T exhibits a significant negative shift. However, when exposed to high-energy X-ray, no shift in V_T is observed, as the transmission rate reaches 100%.

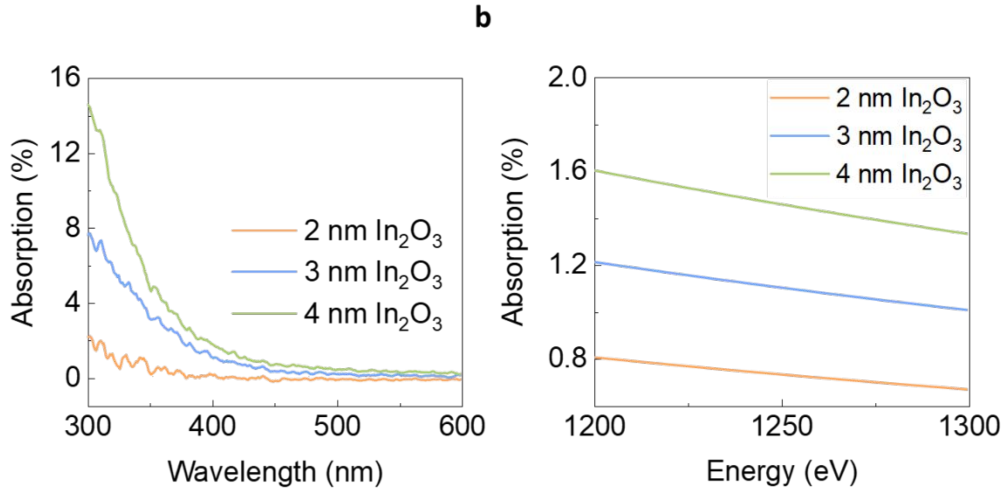

Figure S3. Thickness dependence of In_2O_3 absorption spectra. (a) Optical absorption spectra in the wavelength range of 300 to 600 nm. (b) X-ray absorption spectra (1200 to 1300 eV). The data in (b) were obtained from simulations using the Center for X-Ray Optics (CXRO) database at the Lawrence Berkeley National Laboratory.

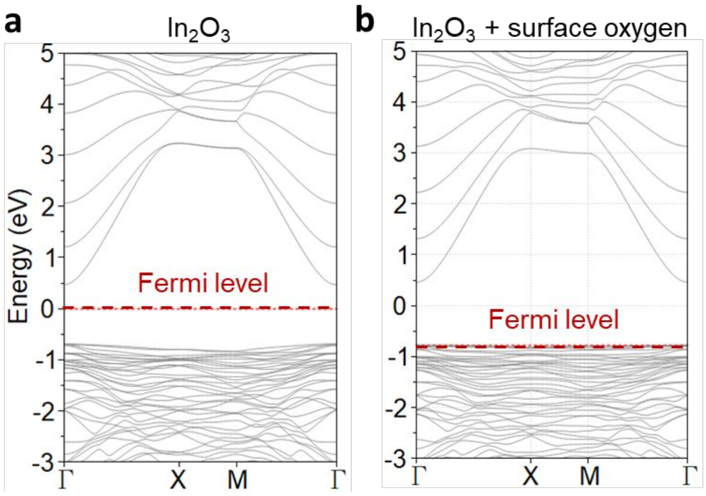

Figure S4. (a) A DFT simulation of intrinsic In_2O_3 surface. (b) A DFT simulation of surface oxygen adsorbed on In_2O_3 surface.

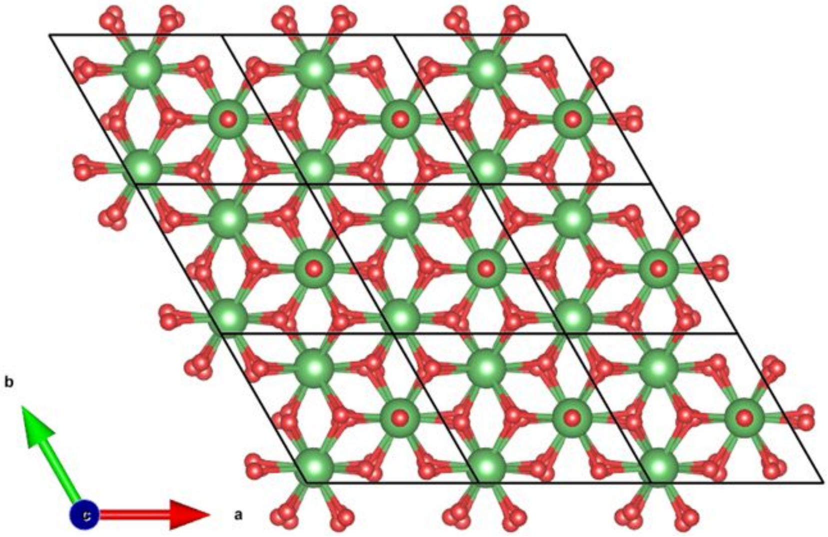

Figure S5. The top view of the optimized structure of the In_2O_3 film used in the DFT calculations. The vectors **a** and **b** of the unit cell were (5.612, 0.000, 0.000) and (-2.797, 4.870, 0.000) in Å, respectively. The indium and oxygen atoms were colored green and red, respectively.

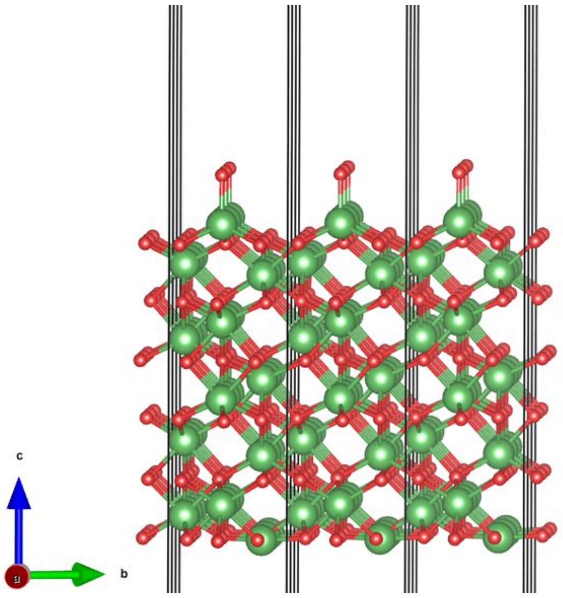

Figure S6. The side view of the optimized structure of the In_2O_3 film used in the DFT calculations. The thickness of the corundum-type In_2O_3 film (without including an O_{sur} atom) was about 14.8 Å in **c**-axis.

Text S2. Time for oxygen diffuse to In_2O_3 surface

We calculate the time required for oxygen to diffuse from the atmosphere to the surface of the In_2O_3. Based on Henry's law, the diffusion of oxygen can be driven by concentration gradients:

$$F_1 = h_g(C_g - C_s) \tag{1}$$

where h_g is gas phase mass-transfer coefficient, C_g is oxygen concentration of air and C_s is oxygen concentration on the surface. The diffusion rate is $9.37 \times 10^{-3} \frac{\text{mole}}{\text{m}^2\text{s}}$ (with the gas-phase mass transfer coefficient is $10^{-3} \frac{\text{m}}{\text{s}}$ and the concentration difference of oxygen is $9.37 \frac{\text{mole}}{\text{m}^3}$). Since the surface area of In_2O_3 is 5×10^{-11} m^2, the amount of oxygen that can diffuse to the surface of In_2O_3 per second is then calculated as $4.7 \times 10^{-13} \frac{\text{mole}}{\text{s}}$. In addition, we deduce that each In_2O_3 device can accommodate approximately 0.3×10^{-15} mole of oxygen. Finally, we can estimate that the diffusion time of the oxygen to the In_2O_3 surface is less than 2 ms.

Text S3. Time for oxygen adsorb on In_2O_3 surface

Oxygen adsorption reaction initiates after diffusing to the In_2O_3 surface. We also calculate the time required for oxygen to adsorb onto the surface of In_2O_3 by the equation:

$$T = \frac{N_{\text{mono}}}{R} \tag{2}$$

The N_{mono} is used to determine the number of molecules needed to form a monolayer on a unit area of a surface and R is the adsorption rate. The N_{mono} is the inverse of the size of oxygen,

which is approximately $1\times 10^{-5}\frac{\text{mole}}{\text{m}^2}$. The adsorption rate is about $9.37\times 10^{-6}\frac{\text{mole}}{\text{m}^2\text{s}}$ (with flux of oxygen molecule at 1 atm is $9.37\times 10^{-3}\frac{\text{mole}}{\text{m}^2\text{s}}$ and the sticking coefficient is 10^{-3}). Finally, the oxygen adsorption time is calculated to be less than 2 s.

Text S4. General solution of two first order reversible reaction

The first order reversible reaction of In_2O_3 surface oxygen charge exchange can be expressed

$$O^-_{sur} + h^+ \underset{k_2}{\overset{k_1}{\rightleftharpoons}} O_{sur} \quad (3)$$

$$O_{sur} + e^- \underset{k_4}{\overset{k_3}{\rightleftharpoons}} O^-_{sur} \quad (4)$$

According to the two equations, the differential equation for concentration over time can be written as

$$\frac{d[O^-_{sur}]}{dt} = -k_1[O^-_{sur}][h^+] + k_2[O_{sur}] + k_3[O_{sur}][e^-] - k_4[O^-_{sur}] \quad (5)$$

Let $[O_{sur}]_{tot}$ be a constant of $[O_{sur}] + [O^-_{sur}]$. We can get

$$\frac{d[O^-_{sur}]}{dt} + (k_1[h^+] + k_2 + k_3[e^-] + k_4)[O^-_{sur}] = (k_2 + k_3[e^-])[O_{sur}]_{tot} \quad (6)$$

Thus, we perform integration on both sides and we obtain the O^-_{sur} concentration as a function of time

$$[O^-_{sur}](t) = [O^-_{sur}]_0\, e^{-(k_1\delta p + k_2 + k_3\delta n + k_4)t} + \frac{(k_2 + k_3\delta n)[O_{sur}]_{tot}}{k_1\delta p + k_2 + k_3\delta n + k_4}(1 - e^{-(k_1\delta p + k_2 + k_3\delta n + k_4)t}) \quad (7)$$

Eq 7 is used to fit the V_T - stimuli time when subjected to stimuli. However, upon removal of the stimuli, the term $[O^-_{sur}]_0$ in **eq 7** requires correction. Upon the termination of the stimuli, as V_T begins to recover, the initial charged surface oxygen should be adjusted from $[O^-_{sur}]_0$ to

$$[O^-_{sur}]_{sat} = \frac{(k_2 + k_3\delta n)}{k_1\delta p + k_2 + k_3\delta n + k_4}([O^-_{sur}]_0 + [O_{sur}]_0) \quad (8)$$

According to **eq 7** and **eq 8**, we can obtain

$$[O^-_{sur}](t) = [O^-_{sur}]_{sat}\, e^{-(k_1 p_0 + k_2 + k_3 n_0 + k_4)t} + \frac{(k_2 + k_3 n_0)[O_{sur}]_{tot}}{k_1 p_0 + k_2 + k_3 n_0 + k_4}(1 - e^{-(k_1 p_0 + k_2 + k_3 n_0 + k_4)t}) \quad (9)$$

Due to the removal of the stimuli, the δn and δp also should be adjusted to n_0 and p_0, respectively. **Eq 9** is employed to model the recovery mechanism by aligning the initial points of the various treatments.

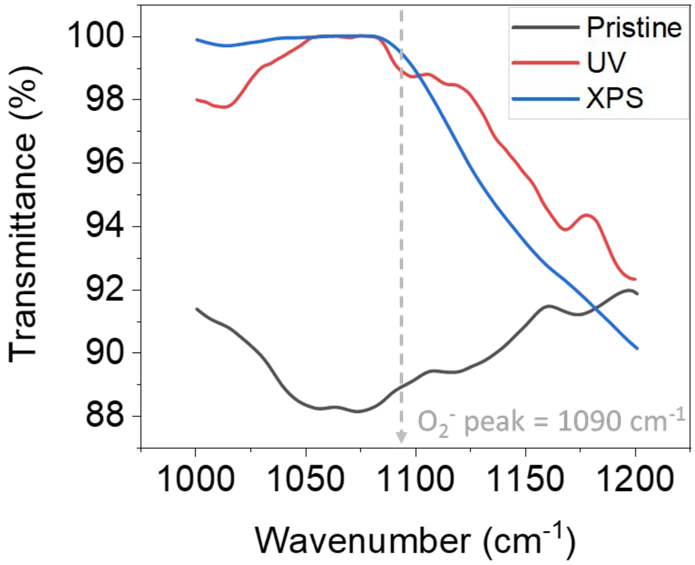

Figure S7. FTIR transmittance spectrum shows the surface-adsorbed oxygen on In_2O_3 films. The increase in transmittance from 88% (pristine) to 96% (UV) and 99% (X-ray) reflects the reduction of surface oxygen concentration through recombination with photogenerated holes.

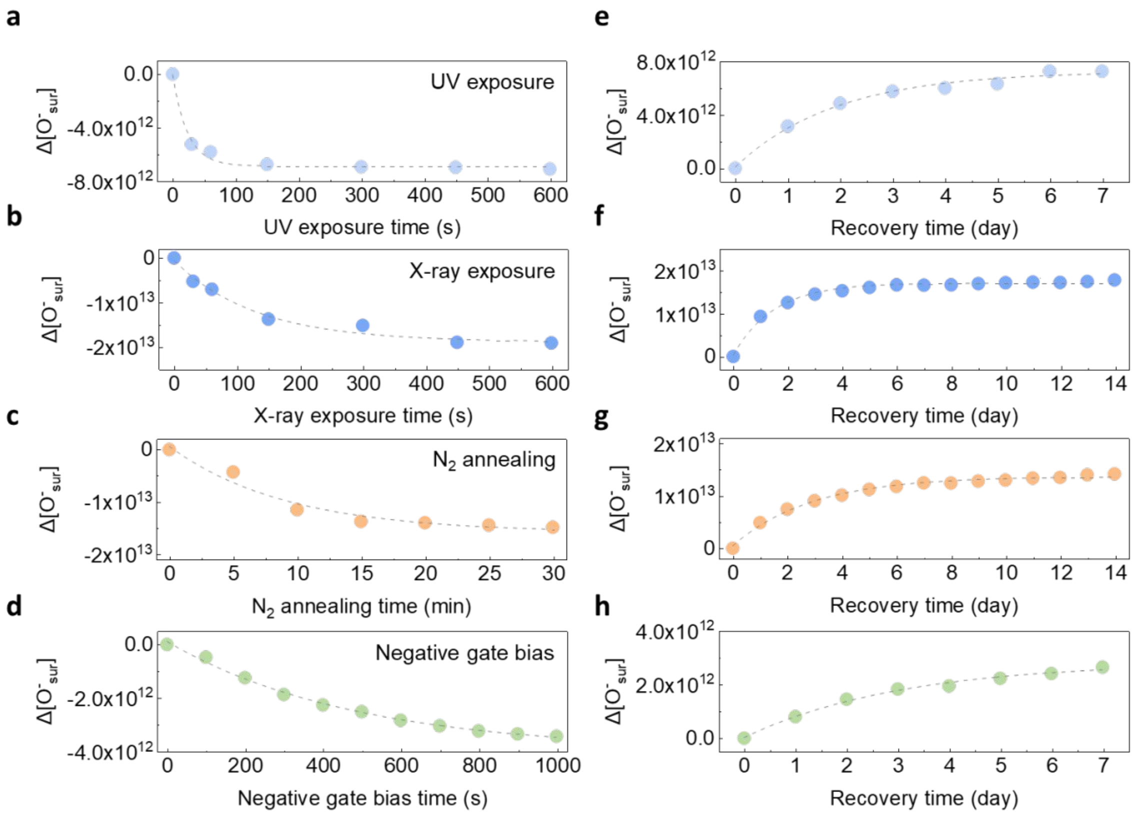

Figure S8. Surface oxygen dynamics under four different perturbation conditions. (a) UV exposure (b) X-ray exposure (c) N_2 annealing (d) Negative gate bias. Surface oxygen recovery

under four distinct perturbations. (e) UV exposure (f) X-ray exposure (g) N_2 annealing (h) Negative gate bias.

Table S1. Comparison of parameters under different perturbation and their recovery

	UV exposure	X-ray exposure	N_2 annealing	NBS
α	- 6.8 x 10^{12}	- 1.8 x 10^{13}	- 1.5 x 10^{13}	- 4 x 10^{12}
τ_1	23 s	130 s	9 mins	480 s
	**Recover from UV**	**Recover from X-ray**	**Recover from N_2**	**Recover from NBS**
β	7.2 x 10^{12}	1.7 x 10^{13}	1.4 x 10^{13}	2.8 x 10^{12}
τ_2	1.9 days	2 days	2.8 days	3 days

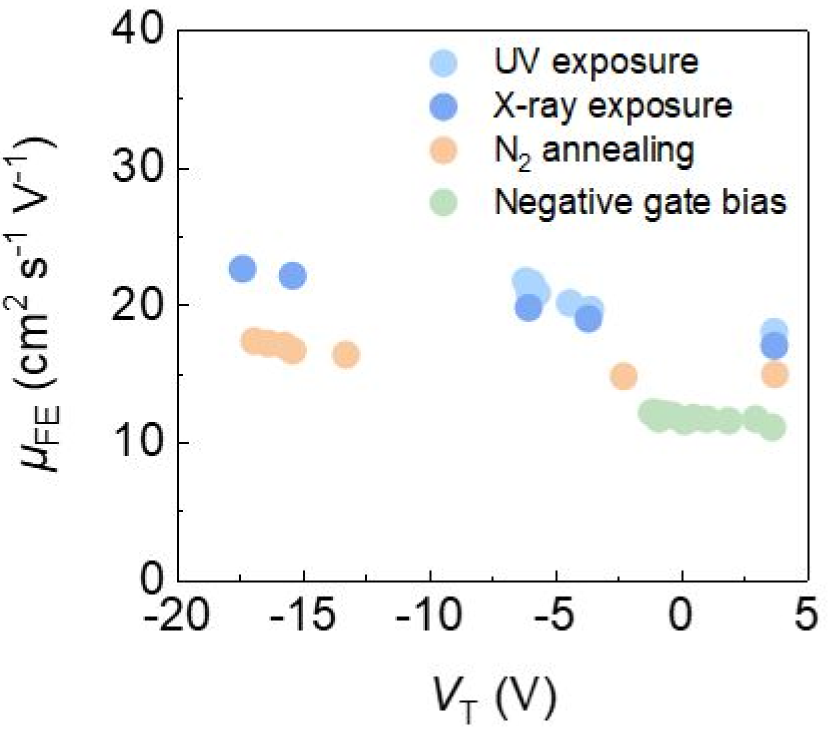

Figure S9. The field effect mobility (μ_{FE}) of ultrathin In_2O_3 transistors as a function of V_T tuned by the proposed methods.

Text S5. Demonstrating the charged surface oxygen concentration equivalence of pre-stimulus and post-recovery states

In the absence of external stimuli, **eq 7** should be modified by replacing δ_n and δ_p with n_0 and p_0, respectively:

$$[O^-_{sur}](t) = [O^-_{sur}]_0\, e^{-(k_1 p_0 + k_2 + k_3 n_0 + k_4)t} + \frac{(k_2 + k_3 n_0)[O_{sur}]_{tot}}{k_1 p_0 + k_2 + k_3 n_0 + k_4}(1 - e^{-(k_1 p_0 + k_2 + k_3 n_0 + k_4)t}) \quad (10)$$

Thus, at t = 0 and t → ∞, the values converge to an equivalent value:

$$[O^-_{sur}]_0 = \frac{(k_2 + k_3 n_0)[O_{sur}]_{tot}}{k_1 p_0 + k_2 + k_3 n_0 + k_4}$$

(11)

Referring to **eq 7** and **eq 9**, when t = 0 in **eq 7**, it should correspond to the asymptotic state of t → ∞ in **eq 9**:

$$[O^-_{sur}]_0 = \frac{(k_2 + k_3 n_0)[O_{sur}]_{tot}}{k_1 p_0 + k_2 + k_3 n_0 + k_4}$$

(12)

This implies that, regardless of the applied stimulus, the system ultimately returns to its initial value.

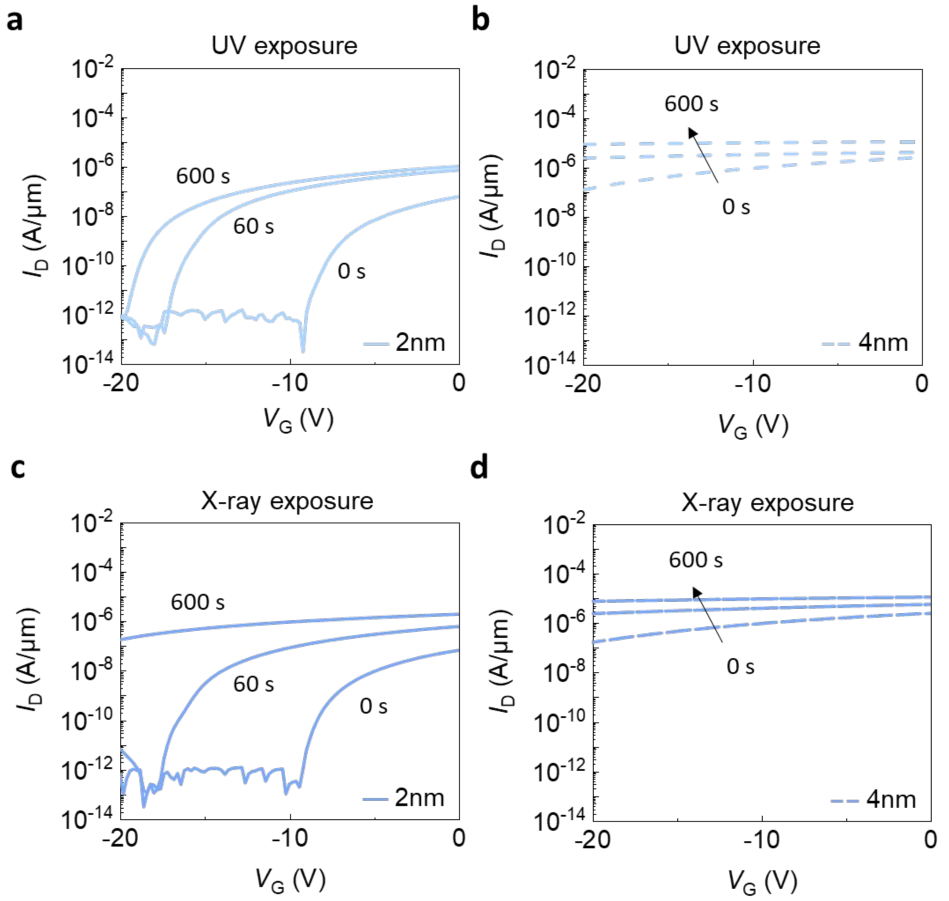

Figure S10. The transfer characteristics I_D−V_G plot of In_2O_3 transistor after different UV exposure time with V_D = 0.1 V. (a) 2 nm (b) 4 nm. The transfer characteristics I_D−V_G plot of In_2O_3 transistor after different X-ray exposure time with V_D = 0.1 V. (c) 2 nm (d) 4 nm.

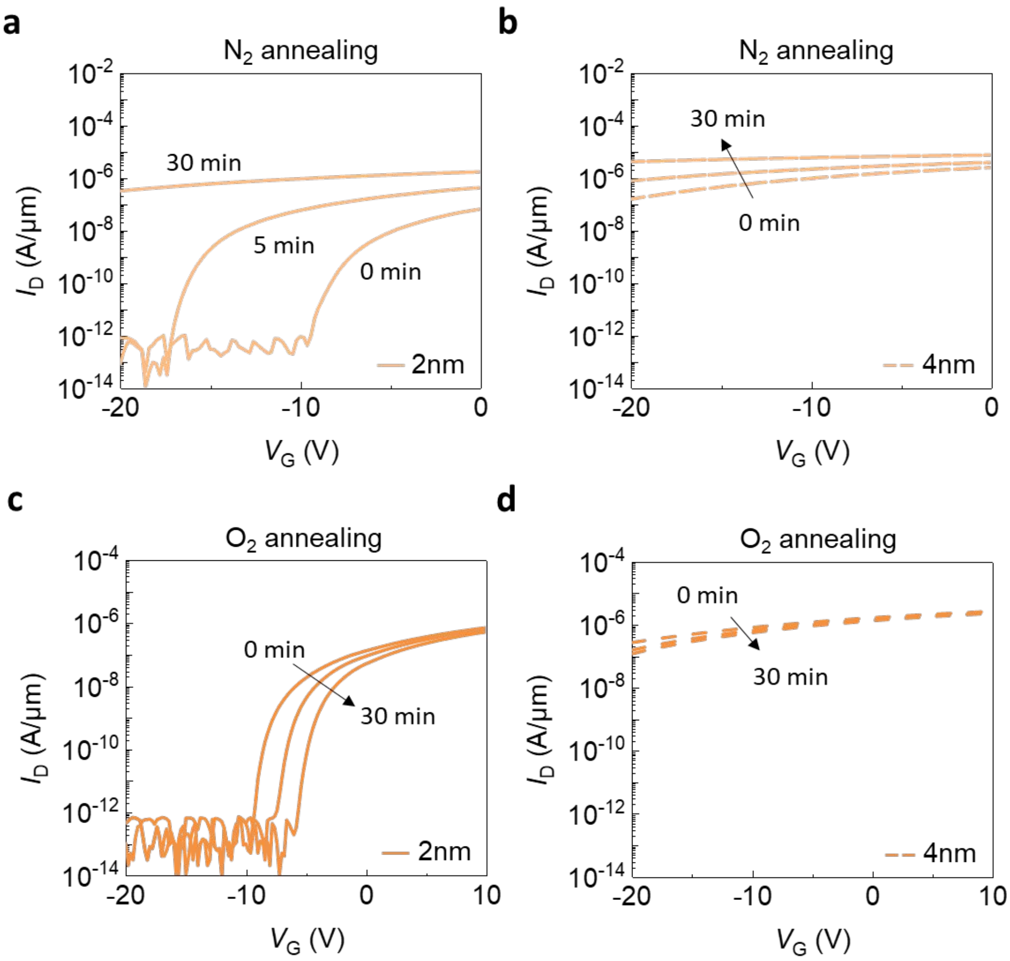

Figure S11. The transfer characteristics I_D−V_G plot of In_2O_3 transistor after different N_2 annealing time with V_D = 0.1 V. (a) 2 nm (b) 4 nm. The transfer characteristics I_D−V_G plot of In_2O_3 transistor after different O_2 annealing time with V_D = 0.1 V. (c) 2 nm (d) 4 nm.

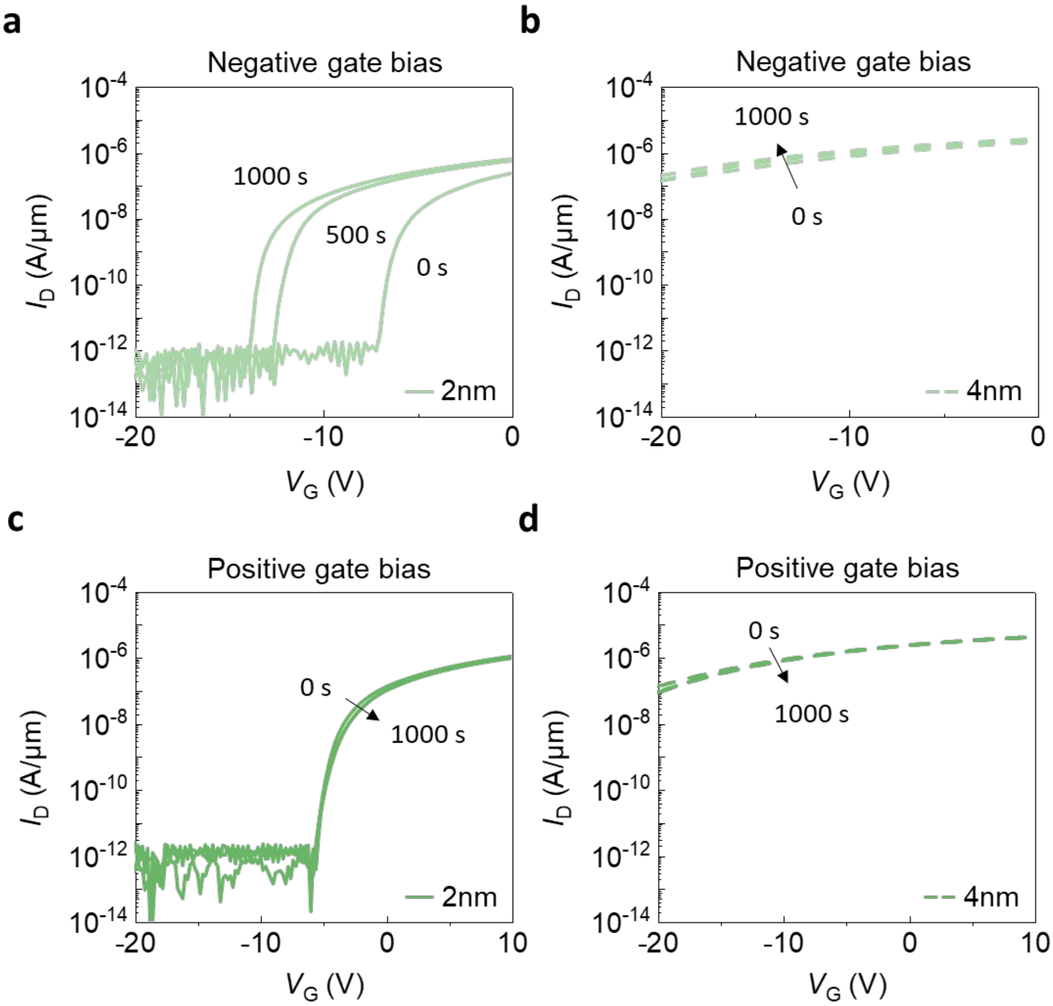

Figure S12. The transfer characteristics I_D−V_G plot of In_2O_3 transistor after different negative gate bias stressing time with V_D = 0.1 V. (a) 2 nm (b) 4 nm. The transfer characteristics I_D−V_G plot of In_2O_3 transistor after different positive gate bias stressing time with V_D = 0.1 V. (c) 2 nm (d) 4 nm.
